# COVID-19 pandemic or chaos time management: first-line worker shortage – a qualitative study in three Canadian Provinces

**DOI:** 10.1186/s12877-022-03419-3

**Published:** 2022-09-03

**Authors:** Idrissa Beogo, Nebila Jean-Claude Bationo, Drissa Sia, Stephanie Collin, Babou Kinkumba Ramazani, Aurée-Anne Létourneau, Jean Ramdé, Marie-Pierre Gagnon, Eric Nguemeleu Tchouaket

**Affiliations:** 1 École des sciences infirmières, School of Nursing, Faculty of Health Sciences, University of Ottawa, Ottawa, ON Canada; 2 College of Nursing, Rady Faculty of Health Sciences, University of Manitoba, Winnipeg, MB Canada; 3 Faculté des sciences de l’éducation, Université Laval, Québec, Québec Canada; 4 Département des sciences infirmières, Université du Québec en Outaouais, Saint-Jérôme, Gatineau, Québec Canada; 5 Département de médecine sociale et préventive, École de santé publique Université de Montréal, Montréal, Québec Canada; 6 École des hautes études publiques (HEP), Université de Moncton, New Brunswick, Moncton, Canada; 7 Canadian Institute for Public Health Inspectors, Vancouver, MB Canada; 8 English Language and Cultural Communities Planning, Programming and Research Officer, Jeffery Hale - Saint Brigid’s, CIUSSSCN, Québec, Canada; 9 Faculté des sciences infirmières, Université Laval, Québec, Québec Canada; 10 Département de Gestion, d’évaluation Et de Politique de Santé, École de santé publique, Université de Montréal, Montréal, Québec Canada

**Keywords:** Older adults, Managers, Frontline workers, COVID-19, Canada, Long-term, Care facilities, Nursing home, Linguistic minorities

## Abstract

**Background:**

Over the successive waves of the COVID-19 pandemic, front-line care workers (FLCWs) —in this case, at long-term care facilities (LTCFs)— have been the backbone of the fight. The COVID-19 pandemic has disproportionately affected LTCFs in terms of the number of cases, deaths, and other morbidities, requiring managers to make rapid and profound shifts. The purpose of this study is to describe the effects of the pandemic on LTCF services offered and LTCFs staff dedicated to linguistic minorities in three Canadian provinces.

**Methods:**

This qualitative descriptive study involved eleven managers and fourteen FLCWs, from six LTCFs of three Canadian provinces (New-Brunswick, Manitoba and Quebec). A qualitative content analysis was performed to identify key themes describing the effects of the COVID-19 pandemic on the services offered and the management of LTCFs staff.

**Results:**

Based on participants’ experiences, we identified three main categories of themes. These macro-themes are as follows: (i) organization and management of staff, (ii) communication and decision-making method, and (iii) staff support.

**Conclusion:**

The study highlighted the tremendous impact of COVID-19 on direct care staff in terms of the high risks associated with caring for LTCFs residents, which are exacerbated by absences and resignations (sometimes up to 50% of staff), resulting in higher resident to FLCWs ratios. Team members had to support each other, they also received accolades and appreciation from the residents.. Finally, the pandemic led to the rethinking of management procedures centred on a coordinated, inclusive and more hands-on management approach.

## Background

The COVID-19 pandemic has profoundly impacted healthcare systems, worldwide. Further, according to [1], the way it has been managed and the significance of its implications to the health system capacity in promoting the population's health was associated with the leadership exercised at a national level. Given the aging population in high-income countries such as Canada [2], long-term care facilities (LTCFs) are a key aspect of the primary care system [3]. The COVID-19 pandemic has highlighted the role of front-line workers at these facilities, namely the nurses and patient attendants—traditionally the backbone of healthcare systems [4]— who provide primary health care [5].

FLCWs include nurses and care givers (herein patient attendants). FLCWs were front and centre in the response to the successive waves of the pandemic, caring directly for infected individuals. Their dedication [6], the development of nursing leadership [7], their increase in turn-over [8], bearing the risk of contracting COVID-19 and infecting family members [9], and the heavy price they paid (e.g., death [10]), led politicians, the media, and the general public to christen them as “heroes” [11].

The severity of the outbreaks and the persistence (multiple waves) of the virus [12], coupled with the historically high mortality rate [13] led to the reorganization of care settings [14]. In fact, the proportion of deaths due to COVID-19 in LTCFs in Canada was twice as high as in other Organisation for Economic Co-operation and Development (OECD) countries [15], for example, 70% in British Columbia between March 1, 2020, and February 15, 2021 [16].

To address these many challenges—all of which are equally urgent— LTCFs managers were forced to adopt a “wartime” management approach [17]. This involved closely managing staff (leaves, resignations, new hires) and equipment, reorganizing clinical activities (Catania et al*.*, 2021), and developing innovative communication methods, both internal (with staff) and external (with family, partners). Faced with so many concerns, they had to find ways to motivate and support the staff, especially the front-line workers, to help them weather the storm and continue providing services [17].

Based on the experience of the hecatomb due to the covid pandemic in Canadian LTCHs, the government took strong research-oriented action, from the very first wave. In response, 22 Implementation Science Teams (IST) of the 10 provinces working with work with Long-Term care and retirement homes, were funded to find the most effective interventions in terms of settings and contexts. These interventions kept in mind the goal of supporting sustainability, advance, and dissemination of promising practices [18, 19]. Finally, the implementation and constant ongoing evaluation of practices and policies will keep residents, families, caregivers, and staff safe from COVID-19 and come out with pandemics preparedness [19, 20].

With each successive wave of the pandemic, management protocols and care mechanisms for LTCF residents were improved and fine-tuned [21]. However, there is very little in the existing literature on the experiences of LTCF staff and how the facilities were run throughout the pandemic. This project on services supplied and LTCF staffing during peak periods of the COVID-19 pandemic, mainly in LTCF of Canadian linguistic minorities settings, intends to inform and build evidence stemmed from personal and first-hand experiences.

## Objective

Describe the effects of the pandemic on the services offered and LTCF staff dedicate to linguistic minorities in three Canadian provinces.

## Methods

### Study setting and design

In Canada, Official language minority communities include Francophones in anglophone provinces or territories (e.g. Province of Manitoba) and Anglophone communities in Quebec (the francophone province). For this pan-Canadian qualitative study, we conducted a convenience sampling of LTCFs in linguistic minority settings. This project targeted all francophone LTCHs in Manitoba, the two anglophone LTCHs in Quebec city, and two francophone LTCHs in NB. Finally, two facilities in New Brunswick (Manoir Edith B. Pinet Inc. and Résidences Lucien Saindon Inc.), two in Manitoba (Villa Aulneau et and Résidence Despins), and two in Quebec (the francophone province,) (Jeffery Hale Hospital and Saint Brigid’s Home) agreed to participate. Participants included managers (*n* = 11) and front-line workers (*n* = 14), including patient attendants and nurses, chosen by convenience sampling.

### Data collection and analysis

#### Data collection

Data were collected from June to December 2021, through the telephone and Zoom, using a sociodemographic questionnaire and semi-structured interview guides. The questions focused on the profile of the LTCF, the challenges related to restricting access to the facility, and the interventions put in place to counter the residents’ social isolation and loneliness. Interviews were conducted by trained research assistants and lasted between 45 and 120 min. They were recorded and transcribed verbatim. Francophone participants were interviewed directly in French and anglophones (of Quebec) in English. Each participant was paid $20.

#### Data analysis

The analysis consisted of describing the participants’ sociodemographic data. The transcriptions were then coded by the research assistant which was then crosschecked by the project coordinator. Throughout the process, they met a total of six times on a weekly basis for approximately 90 min which included follow-up, training, and addressing any emerging concerns. The principal investigator and project coordinator met for two and a half hours every week to discuss findings and any potential issues that may have occurred. All analyses were performed manually. A thematic content analysis was performed, which consisted of identifying verbal or textual expressions and general recurring themes that appeared under various and more concrete contents [22]. The aim was to identify the main themes in order to compare the participants’ responses according to various criteria. The different categories were established based on the interview grid that was used. Throughout the analysis, categories and subcategories were added and adjusted based on the participants’ responses.

The research was approved by the ethics boards of Université de Saint-Boniface, Université de Moncton, the CIUSSS-CN, and University of Ottawa.

## Results

After summarizing the participants’ characteristics, we described the results according to the main themes that were identified from the analyses.

### Characteristics of the participants

Six LTCFs ultimately participated in the study—two in New-Brunswick (NB), two in Manitoba, and two in Quebec—more than half of which were public facilities. One LTCF in Manitoba, Actionmarguerite, withdrew during the data collection phase. Eleven managers and fourteen front-line workers were interviewed. The participating managers were all bilingual and distributed equally across the provinces, i.e., three managers from each province. At least three-quarters of the respondents were men, all of whom have been employed for more than five years. The front-line workers were predominantly women (86%), ages 25 to 63, who have been employed for at least two years.

The characteristics of the participants and the facilities are presented in Table 1.

**Table 1:** Distribution of participants

Facility managers (*n* = 11)		
**Characteristics**	Number (n)	Frequency (%)
**Sex**		
Female	3	27
Male	8	73
**Duration of employment**		
2–5 years	4	36
More than 5 years	7	64
**Facility status**		
Public	3	50
Private for-profit	1	17
Private not-for-profit	2	33
Total	6	100
**Front-line workers (*n* = 14)**		
**Age, years**		
Less than 25	1	7
25–30	4	29
31–40	4	29
Over 40	6	35
**Sex**		
Female	12	86
Male	2	14
**Employment**		
Nurse	5	36
Occupational therapist	2	14
Patient attendant/caregiver	5	36
Secretary	2	14
**Duration of employment**		
Less than 2 years	1	7
2–5 years	6	43
More than 10 years	7	50

### Interview results according to the three main themes

We identified three themes from the interview results: i) staff organization and management, ii) communication and decision-making methods, and iii) staff support.

### Resignations and absence from work

According to several interviewees, there have been numerous resignations at LTCFs. Staff have been dealing non-stop with COVID-19 since the start of the pandemic, and this has led to absences from work and even resignations. However, the extent of these resignations is not the same across all facilities and provinces. For example, while one participant mentioned three or four FLCWs resigning (PL8) from a Quebec City facility, another reported almost half of all FLCWs resigning in the same province: “*Possibly 50% of nurses have resigned…50% of patient attendants, too. I’d say 50%”* (PL4). Another participant from Manitoba reported virtually no resignations at their facility: “*Out of 27 people, only two caught the virus, and they came back to work after their quarantine”* (PL1).

The reasons behind staff resignations or absences vary. Some say it was to protect their family members as a preventative measure: “*No*. *Not because of the pandemic, but for personal reasons, like someone in the family dying or getting sick"* (PL1); “*… Because someone in their family is scared, they’ll bring the virus home. They didn’t do it for themselves—they did it more for their family"* (PL2). Indeed, most nurses and patient attendants either caught COVID-19 or developed symptoms. One manager described the situation well: *“… As I said, we lost about 12 staff members—nurses, housekeepers, patient attendants—and about 15 have caught COVID so far”* (M3).

Most participants recognize that the FLCW staffing crisis created by COVID-19 has greatly impacted the nature and quality of services provided.

### Delivery of healthcare

Repeated absences and resignations have led to changes in the availability and quality of care due to inadequate resident-to-staff ratios. One participant reported situations during the crisis where one nurse may have been responsible for 30 residents (PL7), or nine patient attendants for 50 residents. These employees were unable to adequately meet the residents’ needs (hygiene, food, clothing, etc.). This FLCW said: “Because we don't have a lot of time to…to be with them. You know, like chatting, doing hair, you know, that's the kind of stuff we like… we'd like, but we're short of staff all the time" (PL9)”. According to the FLCWs, while this situation existed long before the pandemic, the arrival of COVID-19 only exacerbated an existing problem, as evidenced by the following comment: “*The pandemic meant we should have had a lot more staff, because we were overloaded*. *There were 5–8 rooms of residents with COVID who needed constant care and temperature checks. Everything was rushed, and we were overworked”* (PL10)*.* The FLCWs also provided crucial insight into physicians’ visits for residents. For instance, PL10 explained how “*the weekly visits of the residents in their rooms by the doctor were impacted by the fact that the doctor came to the facility, but did not enter the residents' rooms, so for the residents it was as if the doctor did not come*” (PL10). PL1 added, in the same vein, that “*the pandemic meant that uh people who went out even to uh get treatment uh they were all stuck inside. They had to see the nurses again and then it became really stressful…*”.

Social interaction also suffered: *“Because we don’t have a lot of time to… spend with them. You know, like chatting, doing their hair… the little things we enjoy and would like to do, but we’re constantly short staffed”* (PL9). These results demonstrated the negative consequences of COVID-19 on the delivery of care by FLCWs. In light of this, steps were taken to reorganize services to deal with this unprecedented situation.

### Services re-organization

To ensure the continuity of services, LTCFs had to be resourceful. Some measures included hiring more staff, reorganizing activities, improving communications, and supporting staff at various levels. For one manager in New Brunswick, this meant consolidating staff by increasing working hours per shift, hiring more employees, and modifying some part-time contracts, for example. According to them, they “*hired new staff and increased everyone’s hours to strengthen the team. Some changes were made by Human Resources… for the residents, we hired, we had a rehabilitation worker who wasn’t full time, so we made them full time*. *We assigned staff to help with activities because we had volunteers”* (M5). Similarly, according to another manager: “*Some ladies were only part time, but now they come in on weekends”* (M7)*.* In Quebec, LTCFs received outside help in the midst of the pandemic, as this manager explains: “*Then, we had the Red Cross come in and help us out*. *The Red Cross, paramedics, too—but they came in maybe two-thirds of the way through the pandemic”* (M8)*.*

The reorganization of services influenced communications and decision-making in LTCFs.

### Bottom-up decision-making model

Communication and decision-making in LTCFs have been forced to evolve over the course of the pandemic. Managers had to make themselves more available, for example through regular meetings*: “We had a meeting every morning where we’d decide: who’s doing what today, and who’s going where”* (M10). Staff were very involved in decision-making and up-to-date information (e.g. clinical guidelines, public health restrictions or vaccination) from public health agencies (provincial or territorial and national) was shared with them systematically. Similarly, the following words from a manager set the tone for the magnitude of the changes and subsequent decisions made at all levels of LTC operations: *"There was the biggest shake-up in the way things were done in a long-term care home, …for all the daily activities, non-health if you want, pi [to means ‘and’] I'm talking about recreation, spiritual services, physical activities, *etc. *(M1)."* For this FLCW from New Brunswick: *“… Despite the fact we’re understaffed, we’ve always managed, if you know what I mean*. *We’re a really great group, with good communication, and things run smoothly… And I’d say the pandemic has made us closer” (*PL10)*.* Likewise, one manager explained how they held more meetings to keep staff informed and up to date on new developments: *“We had a lot of team meetings—meetings with the staff who were there”* (M5). One FLCW added: *“Even (the managers) were there for us when COVID hit hard. They said if we ever had questions or wanted to talk, we should feel free.”* (PL9)*.*

### Staff support and motivation

Psychological support, essential to helping staff cope with the difficult circumstances surrounding the COVID-19 pandemic, took place between staff members or was alternatively provided by managers, residents, and outside resources. The staff felt well supported, which kept morale up and motivated them to keep working. One member of the management staff pointed out that “*the workers needed it*. *I know our managers were there for us, but the support between colleagues is what helped me most, because we were all going through the same thing”* (Eoq). Another added: *“… So, we talked to each other and vented about our experiences. Our managers were there to support us, but at the same time, there was so much to manage that… they weren’t necessarily always available. They were still there, but personally, what helped me most was really… talking about it with my colleagues… supporting one another through it”* (PL6)*.*

There were also incentives offered to staff members, as the following comments show: *“Everyone was encouraged not to miss any days of work, and he (the manager) gave bonuses*. *We heard that in other places, they were getting 2/3 more an hour. We didn’t get 2/3, but we got a bonus”* (PL7*)*. Another participant stated: *“… (the manager) gave out letters, lots of encouragement, lots of thanks—all the time, to everybody.”* (PL2). The residents also encouraged the FLCWs: *“… they hung signs… in the hallways and outside. One of the residents hung thank-you signs everywhere. I came in one day, and I saw thank-you hearts everywhere, and they’re still up. And that’s over a year ago and they’re still on the walls everywhere, that’s beautiful, yeah”* (PL2).

## Discussion

These facilities faced numerous challenges within the scope of management and organization, in terms of both staff and services. Given that, the experiences of FLCWs, who were severely tested during this period, were not always prioritized. The trials and tribulations of FLCWs forced managers to continuously reorganize services in order to cope with the changes brought on by successive waves of the pandemic. Managers had to make decisions quickly, in completely unprecedented situations, and without the benefit of perspective or insight as to what was to come.

Despite efforts to provide services under these extenuating circumstances, management of human resources, including FLCWs, remained one of the biggest challenges, with heavy workloads, medical leaves and staff shortages, and a general feeling of helplessness among the staff [23]. As findings showed, LTCFs focus on essential services, even physicians’ visits decreased or were ultimately cancelled. This may explain why some managers who are typically busy through the day, would set aside their traditional daily tasks and workload to lend a hand to FLCWs. FLCWs felt heart-broken to not be able to address 100% of their resident’s needs, but continue to strive to do their best, while addressing the most essential and basic of needs. There is weak existing literature on such a behaviour but, Ball et al. 2009, working with direct care workers in long-term cares homes, concluded that the FLCWs relationship with their residents matter greatly and are of the primary motive of their overall job satisfaction [24].

This study led to three major findings which include organization and management of staff, the decision-making method, and staff support.

First, the study results show that the COVID-19 pandemic resulted to many medical leaves and even resignations among FLCWs. Several reasons led to this situation. First, considering the significant level of vulnerability, LTCFs are at much higher risk of COVID-19 spread-out and subsequently, FLCWs were disproportionately affected and infected by the virus [25, 26]. Second, as operating staff who are therefore in close contact with patients, FLCWs were at high risk of reporting a positive test for COVID-19, and as a result become both sides vectors of the disease [27, 28]. For these reasons many of them had to call in sick either due to actual illness (sometimes for extended periods of time) or for preventive reasons. As a higher risk population, there was a constant concern about not infecting their family or community members, leading to the often very difficult decision to quit their job, or even to self-marginalize because they felt like they were “part” of the virus [29].

Even in regular times, FLCWs going on medical leave or resigning from their positions at LTCFs has major consequences on services, in particular the minimum worker-to-resident ratio. Instead of the expected increase in ratios in this type of situation, the ratios actually decreased, meaning staff were unable to keep up with regular activities or the task of keeping residents in touch with their families (phone, video, etc.) [30–32]. Many key activities were put on hold or were more difficult to carry out. These include individual and group recreational activities, and baths, as it became harder to spend enough time with the residents to meet their needs. Incidentally, this was already an issue before COVID-19 (Michel, Garcia Manjon, Pasquier, & Ortoleva Bucher, 2021).

The reasons for medical leaves are varied. For example, the automatic removal of sick or symptomatic FLCWs (according to COVID-19 guidelines) was an almost permanent reality, representing one of the biggest challenges for managers [33, 34]. To ensure the continuity of services, managers of LTCFs developed various initiatives, including 1) reinforcing the staff by recruiting agency workers, with the option of long-term contracts (stability, permanence) [35], 2) retraining some managers as FLCWs to assist with personal hygiene, feeding, and dressing the residents.

Second, there was a significant shift in communication and decision-making between LTCFs managers and staff members. Staff were very involved in decision-making, and information was shared with them systematically. All meetings between managers and the FLCWs were an opportunity for improvement decisions (clarification, adjustment, or evaluation). This bottom-up approach [36] helped create a feeling of solidarity between the workers and the managers and increased their sense of belonging during the health crisis.

The situation led to a more participatory type of day-to-day management that not only made workers feel useful but also gave them the confidence and autonomy to do their jobs under very difficult, crisis-level conditions [37]. This bottom-up management approach was a key factor in allowing staff, especially at LTCFs, to continue providing services despite the difficult circumstances. There can be no question the pandemic has taken a psychological, physical, and mental toll on FLCWs (Huerta-González et al*.*, 2021).

Participants reported feeling supported. Beyond the standard support provided by all organizations, during COVID-19, managers doubled down on their efforts to be available to their staff through direct communication and “one-on-one support.” This trend was observed during the study. This strong presence by managers, some of whom did not hesitate to get their hands dirty, is unique, not to mention a significant source of support for the FLCWs, whose workloads were becoming untenable. The residents also witnessed the dedication and self-sacrifice by staff members, showing their support with thank-you signs. As reported in the study by [38] on management of the COVID-19 crisis in France, FLCWs also supported each other by listening to and helping colleagues who felt overwhelmed.

Finally, since not all Canadian provinces and territories were affected by the same waves at the same time, it can be assumed that managers of LTCFs in New Brunswick and Manitoba (where were unaffected by the deadlier first wave) were able to adjust after seeing what happened in Quebec and Ontario [39]. This hypothesis, indeed, was not part of the project. From results, it appears that the burden and hardship experienced by mangers and staffs, were quite similar. As to July 2022, the two most populous provinces (Ontario and Quebec) paid the highest toll (COVID-19 cases and deaths) in total absolute figure but reported per million, the provinces of Manitoba and New Brunswick did not perform far better [40].

### Limitations

The main limitation of this article is that we are presenting preliminary results from a study that is still in the data collection phase, in particular in Quebec. This means we have little information about FLCWs at Quebec facilities that we could use to compare them to FLCWs in the other two provinces. However, we can already get a general understanding of FLCWs’ pandemic experience in long-term care facilities. Once we have the complete results, we will be able to draw a more detailed portrait of the situation.

## Conclusion

Long-term care is one of the sectors hardest hit by the COVID-19 pandemic. This study attempted to shed light on the effects of the COVID-19 pandemic on FLCWs, who are the backbone of the healthcare system, especially LTCFs. Being in direct contact with the residents, they were disproportionately affected by the waves of the virus and were forced to take time off or even quit their jobs for various reasons. This directly affected the quality and delivery of services to residents and led to an urgent need to reorganize services.

Managers at LTCFs had to come up with proactive solutions to continue providing services at these facilities, which were extremely hard hit by the pandemic. These included hiring new staff, adjusting schedules, and even caring for the residents themselves. This reorganization changed the way communication and decision-making take place in LTCFs. Communications became more frequent, even systematic and direct, and staff were involved in the decision-making process. This led to a feeling of solidarity and a greater sense of belonging among staff. Nevertheless, to focus on essential services, hard decision such as reduction of some services (e.g. professional services, social interaction) or merely interrupted (ludic activities) was implemented. Besides, visitation restrictions were an extreme issue to deal with because families remain the major partner of LTCFs.

Finally, during these trying times, FLCWs were able to rely on managers, residents, and their colleagues for support. They felt comforted and empowered to provide crucial services to the residents—the most vulnerable victims of the COVID-19 pandemic.

## Data Availability

The data generated and/or analyzed during this study, which would be needed to interpret, reproduce and develop the results reported in the article, will be made available to the public at the request of the funding institution.

## Abbreviations

CIHR: Canadian Institutes for Health Research
FLCWs: Front-line care workers
LTCFs: Long-term care facilities
TCA: Thematic content analysis
NB: New Brunswick
